# Women’s autonomy in healthcare decision making: a systematic review

**DOI:** 10.1186/s12905-023-02792-4

**Published:** 2023-12-02

**Authors:** Idayu Badilla Idris, Amy Azira Hamis, Ayuzeity Bistari Md Bukhori, David Chan Chee Hoong, Hakimah Yusop, Muhammad Al-Amin Shaharuddin, Nazmeen Adline Fawwazah A. Fauzi, Thinakaran Kandayah

**Affiliations:** Department of Community Health, Faculty of Medicine, Universiti Kebangsaan Malaysia, Jalan Yaacob Latif, Bandar Tun Razak, Kuala Lumpur, Wilayah Persekutuan Kuala Lumpur 56000 Malaysia

**Keywords:** Women, Autonomy, Healthcare, Decision making, Systematic review

## Abstract

**Objectives:**

Although there are calls for women’s empowerment and gender equity globally, there are still large disparities regarding women’s autonomy in healthcare decision making. The autonomy of women is believed to be crucial in improving their health-related outcomes. This review discusses factors that influence autonomy among women in healthcare decision making.

**Design:**

Systematic review.

**Data sources:**

PubMed, Web of Science and Scopus were searched from 2017–2022.

**Eligibility criteria:**

The inclusion criteria include original articles, case studies and reports that has been written in the English Language, while manuscripts with no full article, reviews, newspaper reports, grey literatures, and articles that did not answer the review objectives were excluded.

**Data extraction and synthesis:**

We carried out data extraction using a standardized data extraction form, that has been organized using Microsoft Excel. A narrative synthesis was carried out to combine the findings of all included articles.

**Results:**

A total of 70 records were identified and 18 were reviewed, yielding eight articles to be included in the accepted list of studies. All studies were conducted in developing countries and most of the studies were cross sectional. Factors that were associated with women’s autonomy in healthcare decision making were age, women’s education and occupation, husbands’/partners’ education and occupation, residential location or region of residence, household wealth index as well as culture and religion.

**Conclusions:**

Identification of these factors may help stakeholders in improving women’s autonomy in healthcare decision making. Policymakers play a crucial role in healthcare decision making by enacting laws and policies that protect women's rights, promoting gender-sensitive healthcare services, ensuring access to comprehensive information, promoting health education, and supporting vulnerable populations. These efforts ensure women's autonomy including able to access to unbiased and effective healthcare services.

**Supplementary Information:**

The online version contains supplementary material available at 10.1186/s12905-023-02792-4.

## Strengths and limitation of this study

- We used the PRISMA 2020 checklist to ensure the quality of the study.
- The review covered only English-language materials, hence there is possibility that pertinent items written in other languages were overlooked.
- Other limitations include the diversity and differences in keywords and titles which were used by different researcher that concentrate on similar subjects but the manuscript were disqualified throughout the screening process.
- Only articles from 2017 to 2022 were included, hence reducing the variation in the article searching process.

## Introduction

Research on women’s autonomy in healthcare decision making has been gaining popularity due to its significance in terms of both human rights and healthcare outcomes [1]. One’s autonomy is defined as the individual’s technical, social, and psychological capacity to freely decide on matters pertaining to his or own personal concerns [2]. With regards to autonomy in healthcare decision making, it is one’s ability and freedom to act or make decision for his or her own self and their dependents’ live matters in an unrestricted manner while having unlimited access to relevant information and health care services [3, 4]. However, respecting autonomy is more complicated since most of the people's self-definition and decision-making processes are heavily influenced by the complex social tie-in within their lives. Relationships between two people especially husbands and wives constitute one of the most common types of social ties-ins that may be involved in the process of decision making. This relationship may have an influence on how individual makes decision in various issues, including the provision of medical care [5].

Despite calls for women's empowerment, global disparities persist in women's healthcare decision-making. A study in 57 countries found that sexual and reproductive health decision-making dynamics vary significantly, with 80% in Europe, Latin America, and South-eastern Asia and less than 40% in Middle and Western Africa [6]. Joint decision-making is a crucial aspect of decision-making processes for women, allowing husbands and wives to share consequences and respect their preferences [7]. This approach is particularly beneficial in low- and middle-income countries, where women's healthcare decisions are influenced by external factors, such as social traditions and the cultural context in which they live in, as well as the opinions of their families and communities, and this is especially prevalent in low- and middle-income countries [8].

Women's autonomy significantly impacts health-related outcomes, leading to increased healthcare visits, treatment and adulthood survival [2, 3, 5, 9]. Empowering women in healthcare decision-making and adequate utilization can reduce morbidity and mortality rates in mothers and their children [10]. Therefore, this systematic review was conducted to identify factors that influence women’s autonomy in healthcare decision making.

## Methods

### Research question formulation

The review question was developed based on the PICo (population, intervention/phenomena of interest, context) concept. The PICO (population, intervention, comparator/s, outcomes) framework has been frequently utilised to assess how well a particular treatment is working in terms of how it affects outcomes, while the PICo idea has been proposed for assessing or synthesising expert opinion, text, or policy addressing a certain issue [11]. Based on the PICo concept, population was set as ‘women’; the phenomena of interest were the factors influencing autonomy, and the context was the factors affecting healthcare decision making. The main research question was what are the factors that influence autonomy among women in healthcare decision making.

### Data source and search strategy

**Fig. 1:**
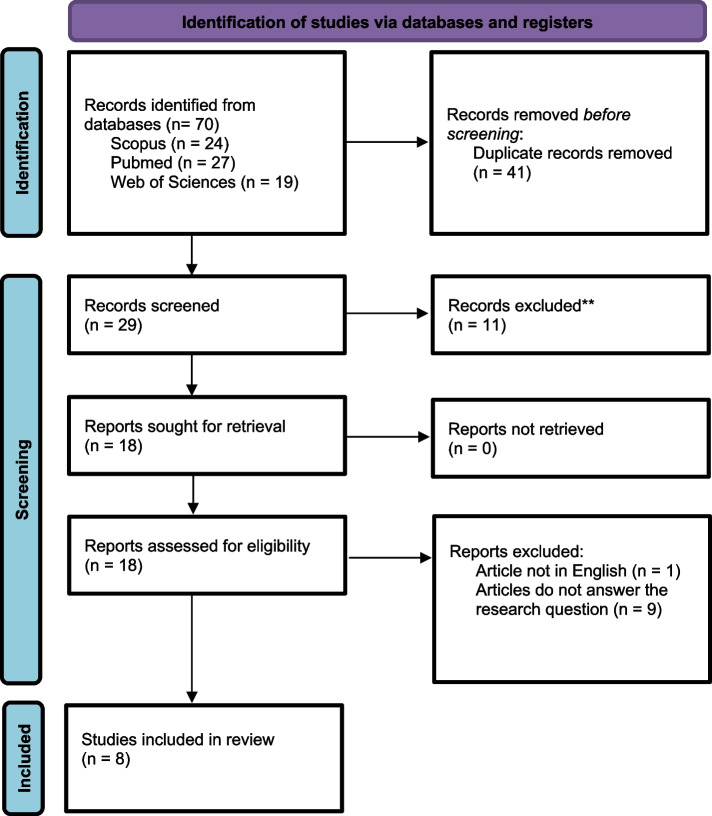
PRISMA flow chart to show the study selection process [12]

During literature search, three databases were included i.e., Scopus, PubMed, and Web of Science. Table 1 lists the keywords that were used to find pertinent articles. The three databases yielded a total of 70 records. A total of 41 duplicate records were eliminated, leaving 29 records for title screening (Fig. 1).

**Table 1:** Keywords search used in the screening process

Database	Search string
Scopus	( TITLE ( women OR female OR ladies OR wife OR wives) AND TITLE ( health OR wellbeing* OR condition* OR wellness) AND TITLE ( autonomy* OR empowerment* OR dependent* OR independent* OR consent* OR freedom OR will*) AND TITLE ( decision* OR choice* OR determine* OR option* OR preference* OR judgement*))
PUBMED	"women"[Title] OR "female"[Title] OR "ladies"[Title] OR "wife"[Title] OR "wives"[Title] AND "decision*"[Title] OR "choice*"[Title] OR "determine*"[Title] OR "option*"[Title] OR "preference*"[Title] OR "judgement*"[Title] AND "autonomy*"[Title] OR "empowerment*"[Title] OR "dependen*"[Title] OR "independen*"[Title] OR "consent*"[Title] OR "freedom"[Title] OR "will*"[Title] AND "health"[Title] OR "wellbeing*"[Title] OR "condition*"[Title] OR "wellness"[Title]
Web of Science	women* OR female OR ladies OR wife OR wives (Title) AND health OR wellbeing* OR condition* OR wellness (Title) AND autonomy* OR empowerment* OR dependent* AND independent* OR consent* OR freedom OR will* (Title) AND decision* OR choice* OR determine* OR option* OR preference* OR judgement* (Title)

### Study selection

The inclusion criteria for study selection were as follows: (i) articles published from 2017 – 2022 and (ii) original articles, case studies and reports. The exclusion criteria were: (i) articles with no full text available; (ii) articles published in languages other than the English Language; (iii) reviews, newspaper reports, grey literatures and (iv) articles that were not related to the main objective which was factors influencing women’s autonomy in healthcare decision making. During the selection process, each of the eight authors screened the titles and abstracts of all the potential eligible articles. In the data conformation process, screened articles were divided randomly among all authors. Each article was then reviewed independently by two authors. Any differences of opinion were addressed by discussions and agreement between the two authors or advice from the study team leader.

### Quality assessment

All included studies underwent a critical evaluation using the Mixed Methods Appraisal Tool (MMAT) [13]. MMAT is an instrument that has been validated for evaluating all study designs, including mixed methods studies [14]. Critical evaluation in this review did not lead to the exclusion of any studies. Each paper was evaluated by two reviewers separately using the MMAT template [13] and their assessments were compared to reach a consensus. Based on the assessment of the study selection bias, study design, data collection methods, sample size, and analysis, the tool assigns a methodological rating of 0, 25, 50, 75, or 100 (with 100 being the greatest quality) to each study.

### Data extraction and synthesis

Data extraction of the included articles was carried out using a standardized data extraction form which was organized using Microsoft Excel. Information collected in these forms include: (1) author and year of publication, (2) references, (3) country, (4) study design, and (5) results or findings.

## Results

In this review, seventy articles were identified from the literature search via three databases, namely SCOPUS, PubMed and Web of Science. Forty-one duplicated records were removed, leaving 29 records for title screening (Fig. 1). A total of 11 articles were removed during the screening, leaving 18 articles for full-text screening. Disagreements were resolved through discussions among the researchers to reach to a consensus. Of the 18 articles, one article was excluded as this article was not written in English, and another nine articles were excluded because they did not answer the research question. After going through careful selection and screening as depicted in the PRISMA flow diagram, only eight articles were included in the full text evaluation (Fig. 1). A descriptive summary of the characteristics of the included studies in this review is shown in Table 2. Similarly, the descriptive summary of findings from the eight studies was included in this systematic review as shown in S1 Appendix.

**Table 2:** Characteristics of the included studies

No	Author (Year), Country	Study design	Participants	Methods
1	Asabu & Altaseb (2021), [2] Ethiopia	Cross-sectional	Women aged 15–49 years old, married and living with husband	Analysis of data from 2005, 2011, and 2016 Ethiopian Demographic and Health Survey (DHS)
2	Alemayehu & Meskele (2017), [4] Ethiopia	Cross-sectional	967 women through three-stage sampling method from 17 rural districts of 2 out of 13 zones of the region	Face-to-face survey through an interview format
3	Osamor & Grady (2018), [5] Nigeria	Cross-sectional	27,135 women aged 15–49 years old who lived with their husbands/partners	Analysis of data from 2013 Nigerian Demographic and Health Survey
4	Ahmed et al. (2019), [9] Pakistan	Q-methodology	60 women, 57 men, 20 healthcare professionals in Lahore, Pakistan	Participants completed Q-sorts individually, and data were collected in person using standardized instructions
5	Mare et al. (2022), [15] Ethiopia	Cross-sectional	3668 married reproductive age women (15–49 y.o) currently using contraceptives	Analysis of data from 2016 Ethiopian Demographic and Health Survey (DHS)
6	Kiani et al. (2020), [16] Iran	Cross-sectional	400 women selected via multistage cluster sampling from attendees of health centers	Data were collected using six questionnaires (demographic, socioeconomic, social support, the Rosenberg self-esteem scale, a marital satisfaction questionnaire, and an empowerment survey)
7	Rizkianti et al. (2020), [17] Indonesia	Cross-sectional	3435 women of reproductive age (15- 49 years) who had given birth within one year preceding the survey	Analysis of data from 2017 Indonesia Demographic and Health Survey
8	Sougou et al. (2020), [18] Senegal	Cross-sectional	8865 women aged 15- 49 years	Analysis of data from 2017 Senegal Demographic and Health Survey

The final eight articles were studies conducted in developing countries, with three studies conducted in Ethiopia, and one each from Nigeria, Iran, Indonesia, Senegal and Pakistan. All studies were cross-sectional except one which was a Q-methodology study. Regarding the type of healthcare decision making, five studies were on reproductive or sexual health decision making while the remaining were on general healthcare decision making.

### Quality assessment

Overall, the eight studies included (100%) scored 100 (high quality studies). There were no “Can’t Tell” or “No” responses noted from the quality assessment of studies.

Several factors have been identified to be associated with women’s autonomy in healthcare decision making, as summarised in Table 3. Five out of the eight articles identified age as one of the factors that influence women’s autonomy and all the papers revealed that, in general, women’s autonomy on healthcare decision making increases with age. Women in the age range of 35 to 49 years, for instance, had greater decision-making autonomy on the use of contraception compared to women in the reference group by more than two times [15]. A study which was conducted in Ethiopia found that women between the ages of 21 and 30 were twice as likely to participate in decision making compared to women under the age of 20, and women beyond the age of 30 were seven times more likely to do so than women under the age of 20 [4].

**Table 3:** Summary of factors associated with women’s autonomy in healthcare decision making

Factors	Authors
Age	Asabu & Altaseb 2021 [2]; Alemayehu & Mesekele 2017 [4]; Osamor & Grady 2018 [5]; Mare et al. 2022 [15]; Sougou et al. 2020 [18]
Women’s education	Asabu & Altaseb 2021 [2]; Osamor & Grady 2018 [5]; Kiani et al. 2020 [16]; Rizkianti et al. 2020 [17]; Sougou et al. 2020 [18]
Women’s Occupation	Asabu & Altaseb 2021 [2]; Alemayehu & Mesekele 2017 [4]; Osamor & Grady 2018 [5]; Kiani et al. 2020 [16]
Husbands’/partners’ education	Asabu & Altaseb 2021 [2]; Alemayehu & Mesekele 2017 [4]; Osamor & Grady 2018 [5]; Kiani et al. 2020 [16]
Husbands’/partners’ occupation	Osamor & Grady 2018 [5]; Kiani et al. 2020 [16]
Household income/Wealth Index	Asabu & Altaseb 2021 [2]; Alemayehu & Mesekele 2017 [4]; Osamor & Grady 2018 [5]; Mare et al. 2022 [15]; Kiani et al. 2020 [16]; Rizkianti et al. 2020 [17]; Sougou et al. 2020 [18]
Residential location/Region of residence	Asabu & Altaseb 2021 [2]; Osamor & Grady 2018 [5]; Mare et al. 2022 [15]; Sougou et al. 2020 [18]
Religion/culture	Asabu & Altaseb 2021 [2]; Osamor & Grady 2018 [5]; Ahmed et al. 2019 [9]; Mare et al. 2022 [15]

Six articles found that the women’s level of education influenced their autonomy on healthcare decision making. A study conducted in Senegal found that women with higher education were 5.5 times more likely to have autonomy on decision making [18]. Meanwhile, another study revealed that uneducated women were 32.2% less autonomous in healthcare decision making [2]. Other study done on women’s autonomy on reproductive decision making showed similar observations [16] and mothers with higher education also showed high participation in healthcare decision making [17].

Women's occupation or employment status was another factor that has been highlighted in four papers as being related to women's autonomy in healthcare decision making. Working women were more likely to participate in healthcare decision making compared to housewife [4] while unemployed women were 45.1% less likely to be autonomous in healthcare decision making. A study conducted in Iran on the autonomy in reproductive decision making also yielded a similar finding [16].

Apart from women’s personal factors, husbands or partners characteristics also played a role in determining women’s autonomy on healthcare decision making. Four studies identified husbands or partners level of education as one of the factors, i.e. women with educated husbands or partners had more autonomy on decision making on healthcare issue [18]. Meanwhile, a study conducted in Ethiopia found that women whose husbands had secondary education or higher were associated with higher autonomy in healthcare decision making [4]. In addition, husbands’ occupation also had an influence on women’s autonomy. This was revealed in a study in Iran which found that husband’s occupation was found to be associated with higher autonomy among Iranian women in reproductive decision making [16]. Women in Nigeria were significantly less likely to decide on their own about their health care if their husbands or partners worked, whether they were professionals or not, than were women whose husbands or partners who were unemployed [5].

Regarding socioeconomic factors, wealth index was cited in seven research as one of the variables that influence women's autonomy in making healthcare decisions. Five studies utilised household wealth index while two studies used asset indicator and women’s wealth index respectively. Majority of these research discovered that there were associations between women's autonomy in healthcare decision making and higher wealth index. However, a single study conducted in Ethiopia gave a different result when women from poor or middle wealth index were more likely to be autonomous compared to those from rich household wealth index [2].

Place of residence, whether urban or rural, were also associated with women’s autonomy in healthcare decision making. Four studies found that women who resided in the urban area were more likely to have higher autonomy in healthcare decision making [2, 5, 15, 18]. In addition, other studies found that living in a specific region was associated with different level of autonomy on healthcare decision making. For example, women who lived in the Addis Ababa city administration, Tigray regional states, and Somali regional states were, respectively, 1.797 times, 1.766 times, and 1.797 times more likely to have higher levels of autonomy in healthcare decision making than women who resided in Dire Dawa city administration. This finding confirmed that the level of women's autonomy was in some way related to region of residence [2].

Four studies also found that religion and culture had a role on the level of women’s autonomy. According to research conducted in Ethiopia, women who were practising Islam, Protestantism, or Orthodox Christianity were less likely to exercise their autonomy than women who practised other religions [2]. Meanwhile, with respect to women’s autonomy on decision making on contraceptive use, research found that Muslim women possessed lower autonomy compared to orthodox women [15]. Culture was also found to be associated with lower autonomy in healthcare decision making. According to the findings of a study carried out in Pakistan, women who made independent decisions were viewed as culturally inappropriate [9].

Finally, there were some other factors that appeared to be associated with women’s autonomy in healthcare decision making. Social support and women’s self-esteem were found to be associated with higher autonomy in reproductive health decision making, while marital satisfaction showed an opposite effect [16]. Those in polygamous marriages were also more inclined to make decisions independently than those in monogamous marriages [5]. Finally, the same study also noted that women who owned their own home were less likely to make decision on their own.

## Discussion

In a variety of health care perspectives, from seeking and utilising medical care to selecting a course of treatment, women’s autonomy has been viewed as significantly necessary in decision making. Female autonomy is important, and in many situations, better health outcomes are associated with their independence in health decision making. The degree on how autonomy is being expressed in different situations relating to healthcare largely depends on several factors including sociodemographic factors as well as other factors.

In this review we selected research on women's autonomy in healthcare decision making which were mainly conducted in developing nations. These studies used a variety of methodologies, and were conducted in various geographic and cultural contexts, involving various health care systems. No study from developed nations was included in this review because female autonomy in developed country was not largely a common issue and it is likely to be well established in these nations.

Results from these studies consistently indicate that women's autonomy in healthcare-related decision-making is positively correlated with age. Older women tend to exert a greater influence and demonstrate higher levels of autonomy in decision-making processes [19]. This may be attributed to their life experiences and past decision-makings that have shaped their independence in healthcare-related decision making. In addition, higher social construct among older women in the society results with higher autonomy. For example, in certain cultural contexts such as in African societies, a women’s perceived social status in the society changes according to their age as well as the roles she assumes [20]. Furthermore, eastern culture believe that older people should be more valued with higher esteem [21]. Another factor contributing to this phenomenon could be due to older women were less afraid to discuss difficulties related to healthcare decision making. As women age, their priorities may shift, and the importance of security and personal satisfaction in healthcare choices may decrease [22].

A structural component linked to women's empowerment in reproductive decision making is women's education, which also has the potential to influence informed decision-making [16]. In this review we found that women with higher education level often had more autonomy in healthcare-related decision making. This finding is aligned with previous studies indicating that autonomy in decision making was associated with women's educational attainment. Attaining at least a secondary education appears to be particularly important in fostering women's autonomy [23]. Educational advancements and new technologies in education may empower women in accessing more information, enhancing their ability to control resources, and fostering decision-making skills. In addition, education may instil feelings of self-worth and self-confidence, which, in turn, may lead to a stronger impact on health-related behaviour compared to mere exposure to pertinent information [22].

Occupation is another important factor which was found to be related to women’s empowerment and has the potential to influence decision making [16]. Studies consistently demonstrate that a woman's level of autonomy was significantly influenced by her employment status [22, 24]. Employed women can have a greater role in the decision-making process, likely due to the positive impact of employment on women’s self-reliance, thus enabling them to actively participate in decision-making [25, 26]. Nevertheless, it is important to note that employment alone may not be sufficient to promote women’s autonomy, as the extent of its influence also hinges on the nature of the work and the associated obligations [27]. When compared with women who were not employed, women in paid employment were more likely to report active participation in making the final decisions [22].

Household income has also been identified as one of the factors associated with women’s autonomy in healthcare decision making. This review confirms a correlation between the household wealth index and women's autonomy in healthcare choices. The ability of women to make independent decisions about their health was higher among those with higher socioeconomic status. It is worth noting that women's income and assets are often intertwined with their educational attainment and employment status. In some regions, men predominantly manage the overall household finances, creating barriers for women in accessing medical care or in accessing transportation to healthcare facilities, which in turn, limits the women's ability to participate in decisions regarding their own health [22]. Conversely, a women’s financial contributions to their families can enhance their value and their status within the household and grant them more negotiating power [28].

Other factors that have been identified to have an influence on women’s autonomy include geographical region, where women who lived in urban area possessed higher autonomy in healthcare-related decision-making process compared to those who lived in rural area. This may be related to the sociocultural attribute among people who lived in rural area. For example, in Africa, patriarchy is often more widespread in rural regions, which lead to higher autonomy amongst male, thus limiting women’s autonomy [29]. Studies also showed that family structure and gender attributes in rural areas can influence women’s autonomy [30]. Likewise, women who lived in rural areas had limited access to education in the community, which would further reduce their autonomy [18].

Religion has also been identified as significantly influence women’s decision-making autonomy. In one study, it was found that Muslim women were less likely to have decision-making autonomy compared to Orthodox religious followers [15], indicating that religion played a vital role in decision-making process [9]. This finding was consistent with the result of other studies which found that there were higher odds of decision-making autonomy on contraceptive use among Christian religion followers [31–33]. Higher levels of autonomy were typically found among women who follow more liberal religious traditions [34]. This may be attributed to the socio-cultural barriers and the respective religious articulation of behaviour [15].

Culture also played a significant role in influencing women’s autonomy in healthcare-related decision making. For example, in Pakistan, it was considered as culturally inappropriate for women to make decisions independently, thus resulting in lower autonomy among women [9]. Another study has described that the traditions related to women’s position in the family varied based on culture in which women must obey their husbands in decision making [35]. This situation may be contributed by the patriarchal culture in the area, which may lead to reduced autonomy among women in making decisions. Within various cultures and tribes, many women had little independence and the power to make choices thus it is important to obtain information on the various contributing factors for decision-making autonomy and disparities across different socio-cultural contexts [4].

Education is another key instrument that may enhance one’s capabilities to adopt new values and transform one's relationships with others in the society. A spouse's education was found to be independently associated with decision making on the topic of sexual intercourse and thus change reproductive health decision-making index [36]. For example, in the decision-making process, husband's education can influence informed decision making. Women with educated husbands had greater autonomy over decision making for their health, as women were supported, and this indicates that family environment had a positive impact on women's decision-making autonomy [18, 37]. Well-educated men exhibit fewer sexist behaviour [38] and were more willing to accept gender equality and believe in equitable decision-making engagement [4]. Educated men may also be more open to alter norms that favour bigger family sizes, and less female empowerment [39].

The empowerment of women involves providing them with increased access to resources, personal control, promoting independence and self-esteem, and enhancing their self-perception. To achieve their reproductive goals, women must be independent, which will enable them to plan their sexual health with greater freedom [16, 40]. Additionally, a substantial increase in the chance of using ineffective contraceptives was observed among women who had low self-esteem. Hence, enhancing self-esteem may have an impact on a woman's ability to make independent reproductive decisions [16], her willingness to refuse undesirable sexual advances, her assertiveness in demanding the use of contraception during sexual encounters, and her ability to talk openly with others about the use of contraception or talking to a healthcare professional [41].

Health is significantly influenced by relationships and social support. One of the barriers to women getting health treatments is lack of social networks. Social support functions as an agent of empowerment and were both a psychological resource and a coping mechanism that results from constructive relationships [42, 43]. However, women's access to practical and emotional social support varies according to socioeconomic status. An absence of sufficient support for women results in employment losses and less community involvement [16, 44].

Women's empowerment in reproductive health decision making has also been significantly associated with marital satisfaction particularly in terms of communication, conflict resolution, sexual engagement, marital cohesiveness, as well as financial planning. Hence, it is thus necessary to build connection between men's participation and women's reproductive health in realising the goal of empowering women [45]. Women's poor health and flawed marital relationships result in low-quality marriages. A study has shown that marital satisfaction influenced the couples’ choice to have their first child [42]. Women were concerned about their health needs in a marital relationship; hence interventions for women's health should be designed to work with families and husbands. Increased women's empowerment through effective spousal communication reduced the risk of failure to meet demands [16].

Women's autonomy in health-related decision-making varies significantly according to individual characteristics, interpersonal, community, and societal levels. It varies across regions and countries, and between developed and developing countries. While the factors influencing women's autonomy, such as education, wealth index and socioeconomic status, religion, culture, and social support, are important in both developed and developing countries, the degree of influence and the specific dynamics can differ. In developed countries, women often have more access to education and economic opportunities, which can enhance their autonomy in healthcare and decision-making processes. Additionally, their culture and religious norms may be more aligned with women's empowerment. In contrast, women in developing countries may have limited access to resources and support systems. They too face greater challenges related to education and socioeconomic disparities. The cultural and religious beliefs may also limit women's autonomy. Overall, the differences in women's autonomy between developed and developing countries are influenced by a complex interplay of these factors.

This review has a few limitations, which include that pertinent articles written in other languages other than the English Language might have been overlooked. Besides, different keywords and titles were used by different research, some of them might have concentrated on similar subjects highlighted in this review but were disregarded during the screening procedure. Finally, the systematic literature extract articles published between 2017 and 2022, hence this may decrease the variation in the article search process.

## Conclusion

In view of the increase in the number of additional task and responsibility faced by women to improve their family health and financial status, women should be given the autonomy to fully exercise their right for healthcare decision making. Husbands or partners should be encouraged to treat their wives with respect and dignity with regular family discussions whilst views and concerns from their wives should not be avoided. Simple behavioural adjustment such as listening could be done and it is imperative for husband or partners to understand that listening is essential in establishing effective communication. The stereotype that husband who listens to the wife will be dominated should be avoided. Hence, opportunity should be given to the wife to express her concern by being a good listener. Apart from that, involvement of various stakeholders such as the health authorities and the non-governmental organisation (NGO) is pertinent to increase the awareness of the importance of women’s autonomy in healthcare decision making among the public. Besides that, prominent public figures such as religious leader, politician, celebrity or even famous national athletes should step forward and increase the awareness on the need for women autonomy in healthcare decision making. This will ensure dissemination of the information reaches the target community effectively.

### Supplementary Information

**Additional file 1: S1 Appendix.** Summary of study findings. **S2 Appendix.** MMAT Checklists.

## Data Availability

All data relevant to the study are included in the article or uploaded as supplementary information.
