# Supplementary material for: Women’s autonomy in healthcare decision making: a systematic review
Source: BMC Womens Health. 2023 Dec 2;23:643. doi: 10.1186/s12905-023-02792-4 (PMC10693143; doi:10.1186/s12905-023-02792-4)
Supplement: Supplementary file 1 — Additional file 1: S1 Appendix. Summary of study findings. S2 Appendix. MMAT Checklists. [file 12905_2023_2792_MOESM1_ESM.docx]

**S1 Appendix. Summary of study findings.**

| **No** | **Author (Year), Country** | **Title** | **Study design** | **Summary/ Results** |
| --- | --- | --- | --- | --- |
| 1 | Asabu & Altaseb (2021) ², Ethiopia | The trends of women's autonomy in health care decision making and associated factors in Ethiopia: evidence from 2005, 2011 and 2016 DHS data | Cross-sectional | Trend of women’s autonomy in health care decision-making had declined. The autonomy of women who resides in urban areas was higher than rural residents, and those who live in the Tigray region, Somali region, and Addis Ababa are higher than who live in Dire Dawa respectively. Unemployed women, women aged from 15 to 24 years, and uneducated women were less likely autonomous in health care decision making. |
| 2 | Alemayehu & Meskele (2017) ⁴, Ethiopia | Health care decision making autonomy of women from rural districts of Southern Ethiopia: A community based cross-sectional study | Cross-sectional | Husband’s education, household wealth index, current age of woman, family size, and woman’s occupation were identified as independent predictors of woman’s health care decision making autonomy. |
| 3 | Osamor & Grady (2018) ⁵, Nigeria | Factors Associated with Women's Health Care Decision-making Autonomy: Empirical Evidence from Nigeria | Cross-sectional | Factors independently associated with decision-making by the woman included: geographical region, rural/urban residence, age, education, religion, wealth index, occupation, home ownership and husband’s occupation. |
| 4 | Ahmed et al. (2018) ⁹, Pakistan | Autonomous decision-making for antenatal screening in Pakistan: views held by women, men and health professionals in a low-middle income country | Q-methodology | Husband and wife should make the decision together and participants strongly agreed with women should not going against their husband’s wishes. It is culturally inappropriate for women to make decisions independently and they should look to what religion says in making decisions. |
| 5 | Mare et al. (2022) ¹⁵, Ethiopia | Married women's decision-making autonomy on contraceptive use and its associated factors in Ethiopia: A multilevel analysis of 2016 demographic and health survey | Cross-sectional | This study revealed that less than one-fourth of married women of reproductive age (15–49 years) had decision-making autonomy on contraceptive use. Place of residence, community exposure to family planning message, women’s current age, age at first marriage, religion, exposure to mass media, household wealth index, and visiting health facility were identified as the factors affecting women’s decision-making autonomy on contraceptive use |
| 6 | Kiani et al. (2020) ¹⁶, Iran | Structural equation modeling of psychosocial determinants of health for the empowerment of Iranian women in reproductive decision making | Cross-sectional | The Iranian women had an average level of empowerment with respect to reproductive decision making. Factors such as women’s education, men’s education, women’s occupation, men’s occupation, asset indicator, self-esteem, and social support are correlated to women’s empowerment in reproductive decision making. |
| 7 | Rizkianti et al (2020) ¹⁷, Indonesia | Women's decision-making autonomy in the household and the use of maternal health services: An Indonesian case study | Cross-sectional | Women with primary education mostly had no participation in decision-making compared to those with higher education level, as they tended to have a higher participation level. Women coming from households with higher wealth status tended to have a higher WPI score. |
| 8 | Sougou et al. (2017) ¹⁸, Senegal | Women's autonomy in health decision-making and its effect on access to family planning services in Senegal in 2017: a propensity score analysis | Cross-sectional | Only 6.26% of women had the latitude make their own decisions about their health.  Women’s autonomy over decision-making increases with age. Women living in rural areas have less autonomy over decision-making for their health. Women with a higher level of education are 5.5 times more likely to have autonomy over decision-making. Richer and wealthier women have more autonomy over decision-making. Women in unions (cohabitation) have more autonomy over decision-making. Women whose partners/husbands are educated have more autonomy over decision-making for their health. Educated women also have more autonomy over decision-making. |

**S2 Appendix. MMAT Checklists.**

| **Authors** | **Screening Questions** | | **Quantitative Descriptive Studies** | | | | | **MMAT scoring** | **MMAT Rating** |
| --- | --- | --- | --- | --- | --- | --- | --- | --- | --- |
|  | S1. Are there clear research questions? | S2. Do the collected data allow to address the research questions? | 4.1. Is the sampling strategy relevant to address the research question?  (Yes=1 /No=0 /Can’t tell=0) | 4.2. Is the sample representative of the target population?  (Yes=1 /No=0 /Can’t tell=0) | 4.3. Are the measurements appropriate?  (Yes=1 /No=0 /Can’t tell=0) | 4.4. Is the risk of nonresponse bias low?  (Yes=1 /No=0 /Can’t tell=0) | 4.5. Is the statistical analysis appropriate to answer the research question?  (Yes=1 /No=0 /Can’t tell=0) |  | 75-100 = High quality  50 – 75 = Moderate quality  Less than 50 = low quality |
| Asabu & Altaseb 2021 | Yes | Yes | Yes | Yes | Yes | Yes | Yes | 5/5= 100 | High |
| Rizkianti et al. 2020 | Yes | Yes | Yes | Yes | Yes | Yes | Yes | 5/5 = 100 | High |
| Alemayehu & Mesekele 2017 | Yes | Yes | Yes | Yes | Yes | Yes | Yes | 5/5/ = 100 | High |
| Osamor & Grady 2018 | Yes | Yes | Yes | Yes | Yes | Yes | Yes | 5/5 = 100 | High |
| Kiani et al. 2020 | Yes | Yes | Yes | Yes | Yes | Yes | Yes | 5/5 = 100 | High |
| Ahmed et al. 2019 | Yes | Yes | Yes | Yes | Yes | Yes | Yes | 5/5 = 100 | High |
| Mare et al. 2022 | Yes | Yes | Yes | Yes | Yes | Yes | Yes | 5/5 = 100 | High |
| Sougou et al 2020 | Yes | Yes | Yes | Yes | Yes | Yes | Yes | 5/5 = 100 | High |
